# A Self-Timed Multipurpose Delay Sensor for Field Programmable Gate Arrays (FPGAs)

**DOI:** 10.3390/s140100129

**Published:** 2013-12-20

**Authors:** Carlos Gómez Osuna, Pablo Ituero, Marisa López-Vallejo

**Affiliations:** Dpto. de Ingeniería Electrónica, ETSI Telecomunicación, Universidad Politécnica de Madrid, Avenida Complutense 30, Madrid 28040, Spain; E-Mails: cgosuna@yahoo.com (C.G.O.); marisa@die.upm.es (M.L.-V.)

**Keywords:** sensor, delay, temperature, FPGA, monitoring, PVT variations, clockless, aging, self-timed

## Abstract

This paper presents a novel self-timed multi-purpose sensor especially conceived for Field Programmable Gate Arrays (FPGAs). The aim of the sensor is to measure performance variations during the life-cycle of the device, such as process variability, critical path timing and temperature variations. The proposed topology, through the use of both combinational and sequential FPGA elements, amplifies the time of a signal traversing a delay chain to produce a pulse whose width is the sensor's measurement. The sensor is fully self-timed, avoiding the need for clock distribution networks and eliminating the limitations imposed by the system clock. One single off- or on-chip time-to-digital converter is able to perform digitization of several sensors in a single operation. These features allow for a simplified approach for designers wanting to intertwine a multi-purpose sensor network with their application logic. Employed as a temperature sensor, it has been measured to have an error of ±0.67 °C, over the range of 20–100 °C, employing 20 logic elements with a 2-point calibration.

## 1. Introduction

The benefits of the extreme technology scaling achieved in current electronic circuits are jeopardized by process, voltage and temperature (PVT) variations along with wearout [1]. Process fluctuations introduce both die-to-die correlated variations and intra-die random variations that undermine circuit performance. With increasing power demands, power supply voltages are becoming more and more susceptible to IR and L*dI*/*dt* drops. Also, compaction of logic in the nanometer regime translates into increased power densities that produce elevated on-chip temperatures. Aging phenomena like hot-carrier effect, time-dependent dielectric breakdown (TDDB), electromigration, thermal cycling, stress migration, and bias temperature instability (BTI) are a growing issue as the integration levels continue to increase at a rapid pace. An interesting approach to fight against all these effects is to employ embedded monitors that either on- or off-line characterize the variation sources so that the necessary design or adaptation is carried out [2–4].

Nowadays, FPGAs (Field Programmable Gate Arrays) represent one of the most important engines of the microelectronics market. Their high demand and sales volume make FPGA vendors constantly adapt their architectures to the latest technology nodes. This means that these devices are very sensitive to PVT and aging variations [5]. Due to their reconfigurability, FPGAs offer a unique opportunity for tailored monitoring and characterization under varying scenarios.

Delay and ring oscillator-based sensors are the most common way to obtain operational information and measure temperature variations in a programmable device [2]. These sensors employ the same logic building blocks used for application programming to obtain relevant data in an environment where very little or no other sensing capabilities exist. Measurements are obtained through the observation of a known circuit topology (the sensor) under a set of operational conditions and estimating the value of the parameter of interest. For example, there are works that take advantage of the existing correlation between combinational delay and operational temperature (which present a quasi-linear relationship) [6,7]. The output of these sensors is usually processed by a time-to-digital or a frequency-to-digital converter, which controls the input and samples the output of the sensor at a high enough frequency to achieve the required accuracy.

This paper presents a novel self-timed multi-purpose delay sensor for FPGAs which, through the use of asynchronous logic, carries out a delay measurement without the need of an external clock. Specifically, the sensor generates a pulse whose width is the amplification of the delay of a signal going through a delay-chain. The proposal displays the following advantages:

- It rests load to the clock trees, one of the scarcest resources in the FPGA. This simplifies the routing process and avoids the complexity of having to work with multiple clock signals. Also, clock-gating policies, when the sensors are not used, are no longer required.
- The time-to-digital conversion can be realized either on- or off-chip. A single converter can be employed to perform several digitizations at the same time, reducing area and power overheads. Furthermore, the communication of the sensor measurement to the converter just requires a varying-width pulse, which is a very efficient signal from the power perspective. Any type of noise induced by the time-to-digital converter—such as self-heating, in the case of temperature sensing—is taken far from the observation point.
- The fact of not needing an external clock improves the sensitivity of the sensor, since its measuring ability will only be limited by the timing of underlying fabric. It will be the frequency employed at the converter which introduces the quantization error.

The proposed sensor has been validated and characterized to measure process and temperature variations. When employed as a temperature sensor, it has been measured to have an error of ±0.67 °C, over the range of 20–100 °C, employing 20 logic elements with a 2-point calibration.

The rest of the article is organized as follows. Section 2 reviews the related literature on FPGA sensing. Section 3 describes the proposed sensor in detail. Characterization and comparison data for the sensor both for constant temperature and varying temperature are put forward in Section 4. Finally some conclusions are drawn in Section 5.

## 2. Background

Instantiating sensors to measure different magnitudes inside an FPGA is not an easy task because FPGA manufacturers leave little freedom to designers beyond standard digital architectures. The high degree of automation achieved in the FPGA design flow comes at the price of increasing the difficulty to manually alter the underlying circuitry to build a certain sensor. However, although on-chip voltage and current data are still out of reach, the scientific community has been able to extract information from a different magnitude: the delay. The time it takes for a signal to traverse a block of logic, a transmission line or a combination of both has a well-known response to different magnitudes, thus it serves well as a sensor. Normally, delays inside the FPGA are very short, therefore, in order to be digitized, they require a time-to-digital converter fed by very high frequencies. A common solution is to use a feedback system that makes the signal oscillate through the delay chain, what is known as a ring oscillator, in this case the digitization is performed by a frequency-to-digital converter, which is normally a counter clocked by the oscillating signal.

Ring oscillators as temperature and variability sensors have already been studied to a great extent. In [8], ring oscillators are studied as a means to monitor temperature in an FPGA, constructing a quadratic frequency versus temperature sensor transfer function, and also studying how voltage variations affect the sensitivity of a sensor, as a function of the number of stages in the oscillator. In their work, they find that for longer oscillating chains, voltage variations are evened-out. Other studies, such as [7], make use of ring oscillators to estimate within-die variability, temperature, and also to observe the power consumption distribution within the die by obtaining readouts while the onboard application is active and when it is idle.

Delay sensors have also been proposed for measuring temperature variations on an FPGA [6]. These sensors comprise a clocked pulse generator, a delay chain and a counter/comparator clocked by the pulse injected in the delay chain, using the counter/comparator block to amplify the time of the delay chain without sacrificing accuracy. Pulses of a fixed length are fed into the combinational chain, whose delay is dependent on operational conditions. The pulse injected into the delay chain is used to clock a circulation counter/comparator pair which instructs the pulse generator to feed yet another pulse to the delay chain, until a certain count is reached. This design amplifies the total combinational delay, enhancing the accuracy of the sensor while keeping a low area.

All previous topologies make use of pulse generation mechanisms to avoid the pulse filtering effects introduced by unevenness in the construction of gates in a delay chain. These pulse generation mechanisms rely on a system clock signal to provide with a fixed length (usually of a few system clock cycles) pulse at the input of the delay chain. While the usage of a system clock signal solves pulse filtering effects, it also limits the performance of the sensor, since total response time will be limited by system clock frequency and by the selected length of the pulse. Additionally, using multiple clock signals in a given block may also pose a problem when automating the deployment of sensors with different delays.

Our proposal also employs time amplification of a delay chain, achieved by feedback and repetition count—which might also be thought of as a kind of ring oscillator—without the need of any external clock. Thanks to a new design of the pulse generating logic, the timing response of our proposed sensor is only limited by the minimum timing requirements of the device under observation. The sensor output is just an analog signal represented by the width of a pulse. The digitization can be performed far from the observation point at very low communication costs, which can be beneficial for some magnitudes—e.g., when measuring temperature time-to-digital conversion can add extra temperature through self-heating.

Taking the ideas presented in [3] and using their network topology, several sensors can share the same time-to-digital converter, which just requires one operation for all the sensors as the start of the measurement is synchronized for them all. Furthermore, as explained in [3], the prioritizing of the sensors' data is performed in a very straightforward way as fastest measures (with least temperature, or fastest process corner, or least aged circuitry) come first and the rest come ordered as the end of their pulses arrives at the converter.

## 3. Sensor Overview

**Figure 1.**
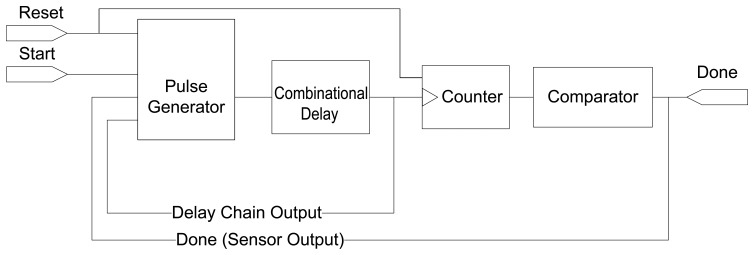
Sensor structure.

The sensor conceptual block-diagram is shown in Figure 1, which includes a pulse generator, a delay chain and a circulation counter/comparator. The interface of the sensor is defined by two inputs, Reset and Start, and a single output, Done. The delay chain acts as the sensing device. The counter is locally clocked by the pulses sent by the pulse generator whose frequency depends on the combinational delay chain. When the count reaches a predefined value, the comparator sets the Done signal high. The time interval between the Start signal and the Done signal edge is the sensor measurement which will be retrieved by the interfacing logic. The length of this interval displays predictable responses under varying temperature, aging and process fluctuations. An external time-to-digital converter is required to perform the digitization of the measurement.

**Figure 2.**
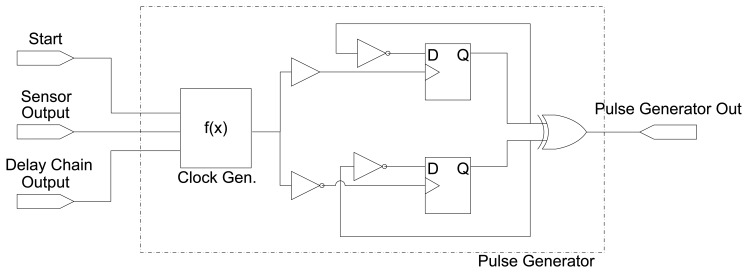
Pulse generator structure.

The key element of the sensor is a self-timed structure built from the pulse generator depicted in Figure 2. This unit comprises two D-FFs, both clocked by the output of a combinational function of three inputs, *f*(*x*): Start, Done, and the output of the Delay element. This function employs combinational logic to generate a local clock signal whose width is dependent on the external delay chain, and allows to reset and set this signal by means of the Start and Done inputs. In order to fully generate the delay pulse, one of the D-FFs is triggered by the rising edge of the clock generator function, and the other one by its falling edge. Table 1 shows the truth table for the clock generator combinational function.

Timing equalization between the clock signals for both D-FFs is achieved by controlling the placement of all logic and memory elements involved. This can be achieved making use of the constraints supported by the FPGA vendor place&route (P&R) tools, a process which can be automated through configuration scripts.

**Figure 3.**
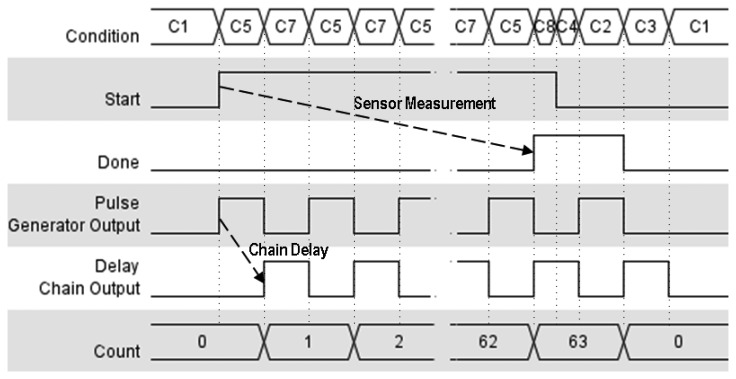
Chronogram of the main signals involved in the self-timed sensor.

Figure 3 shows the functioning of the main signals generated in the self-timed structure. Note that this is an asynchronous circuit and transitions between conditions depend on the involved combinational delays.

In detail, the clock generator function would remain idle as long as Start is low (condition C1), activating the sensor as soon as a rising edge is seen on this port (condition C5). Once the first pulse has been sent through the delay chain, it is fed back into the clock generator to send the corresponding new pulse. The pulse generator would remain clocking pulses through the delay chain and into the clock of the counter (*i.e.*, cycling from condition C5 to condition C7) until the count limit—63 in Figure 3—is reached and the comparator signals that the sensor has finished its measuring time, activating Done (condition C8). Once Done is high, the clock generator combinational function will ensure that:

- The clock signal is pulled low (non-active edge for both D-FFs) and no further action is taken until the sensor has received the Done signal and has pulled Start low accordingly (condition C8 to condition C6).
- As soon as Start is low, one further clock cycle is generated by this function, *f*(*x*), to enable the counter to reset and pull Done low again (conditions C6, C2, C3 and finally C1).

This combinational logic has been carefully designed such that it is guaranteed that there is no possible glitch in the clock generation function. Even considering unexpected conditions due to noise, the output of *f*(*x*) is always glitch-free.

As far as the delay chain is concerned, it is similar to that described in [6], using the LUTs and D-FF/Latches present in the FPGA. Both location and length of the delay chain can be automatized using FPGA P&R tools. As sensing element, it is fundamental to properly analyze and characterize the behavior of this delay chain, as will be done in Section 3.1.

The proposed sensor setup timing aspects are only limited by the switching characteristics of the underlying fabric. The critical timing limitation in this case is ensuring that the pulse created by the pulse generator and the delay chain complies with FPGA minimum clock pulse and setup times. The length of a pulse will be given as the sum of the total delay introduced by the chain of delay elements, plus the delay of the pulse generator and plus routing delay. Since the pulse generator is a fixed structure, the only choices to be made by the designer are the length of the delay chain and the value for the count limit. The sensor accuracy can be thus controlled by the delay chain length and the count limit; therefore there is a trade-off between the sensor accuracy and sampling frequency.

### 3.1. Analytical Description

This section introduces the analytical description of the combinational delay block which controls the complete sensor response. The analysis departs from the detailed path model delay presented in [9], which, apart from wire delay, just considers two transistor-level primitives to construct combinational delays: the inverter and the pass transistor. The propagation delay, *t_drc_*, of wire with a distributed resistance, *R* and capacitance, *C*, is given by [10]:

(1) $$t_{\text{drc}}=0.38\;RC$$

It is known that the delay of a short-channel CMOS logical inverter, *t_inv_*, with balanced rise and fall times, is governed by its physical features according to the following simplified model [10]:

(2) $$t_{\text{inv}}=0.52\frac{(L/W)C_{L}}{\mu C_{OX}\;V_{\text{DSAT}}}$$

where *C_l_* is the load capacitance; *C_OX_* is the gate oxide capacitance; *L*/*W* is the aspect ratio of the N transistor; *μ* is the carrier mobility and *V_dsat_* is the saturation drain voltage. In the case of a network of *n* pass transistors, the delay, *t_npt_*, employing the Elmore approximation, is given by [10]:

(3) $$t_{\text{npt}}=0.69C_{L}R_{eq}\frac{n(n+1)}{2}$$

where *R_eq_* is the equivalent resistance of the pass transistor gate which is inversely dependent on the current it yields.

Employing the nomenclature established in [9], our delay line goes through a number of logic clusters—comprising both logic elements and a local routing crossbar—and several switch boxes that connect the logic clusters. All these can be modeled as a set of m*_inv_* inverter equivalent circuits; *m_npt_* chains of pass transistor equivalent circuits and *m_drc_* distributed RC wires; the total delay, *t_tot_*, is given by the following expression:

(4) $$\begin{array}{ll} t_{\text{tot}} & =\overset{m_{\text{inv}}}{\underset{i=1}{\text{∑}}}t_{\text{invi}}+\overset{m_{\text{npt}}}{\underset{i=1}{\text{∑}}}t_{\text{npti}}+\overset{m_{\text{drc}}}{\underset{i=1}{\text{∑}}}t_{\text{drci}}\\ & =\overset{m_{\text{inv}}}{\underset{i=1}{\text{∑}}}\frac{(L_{i}/W_{i})C_{Li}}{\mu C_{OX}\;V_{\text{DSAT}}}+\overset{m_{\text{npt}}}{\underset{i=1}{\text{∑}}}0.69C_{Li}\;R_{\text{eqi}}\;\frac{n_{i}(n_{i}+1)}{2}+\overset{m_{\text{drc}}}{\underset{i=1}{\text{∑}}}0.38R_{i}C_{i} \end{array}$$

Once we have developed this model for the delay under nominal conditions, let us analyze its behavior under other scenarios, specifically process and temperature variations.

Concerning the former, in [11], a simple statistical model is used to characterize both systematic and random contributions to the actual delay of a circuit element. According to them, any delay path will be made out of a systematic delay variable and a random delay variable, both of them with a Normal distribution. To this simple model, even further simplifications can be applied for closely placed elements. As shown in [12,13] a high degree of correlation can be expected between elements located within the same mm^2^. This implies that for sufficiently close delay elements the systematic delay value will be approximately constant. Applying the model and simplifications from [11], the expression of the total delay considering process variations is given by:

(5) $$t_{\text{tot}}=\sum_{i=1}^{m_{\text{inv}}}t_{\text{inv}i}+\sum_{i=1}^{m_{\text{npt}}}t_{\text{npt}i}+\sum_{i=1}^{m_{\text{drc}}}t_{\text{drc}i}+N(0,\sum_{i=1}^{m_{\text{inv}}}\sigma_{\text{inv}i}^{2}+\sum_{i=1}^{m_{\text{npt}}}\sigma_{\text{npt}i}^{2}+\sum_{i=1}^{m_{\text{drc}}}\sigma_{\text{drc}i}^{2})$$

where *σ* refers to the variance of the random delay component for each element and *N* is the normal distribution. As shown, the total delay effectively tracks the timing fluctuations produced by process variations and can be employed to help designers calibrate their devices.

As long as temperature is concerned, in Equation (2) carrier mobility *μ* is affected by the operating temperature, according to:

(6) $$\mu (T)=\mu_{0}{\left(\frac{T}{T_{0}}\right)}^{-k\mu }$$

where *μ*_0_ is the mobility at room temperature *T*_0_ and *k_μ_* is a fitting parameter generally in the range of 1.2–2.0. Also, in Equation (3)*R_eq_* is inversely proportional to the current of the pass transistor, *I_d_*. This current, operating in any of the transistor on states, is directly dependent on the carrier mobility, *i.e.*, *I_d_* ∼ *μ*(*T*), thus we can conclude that *t_npt_* ∼ 1/*μ*(*T*). From the last expression and Equation (2), it can be seen that the effect of temperature on both transistor-level primitives is dominated by carrier mobility in a quasi-linear way (depending on the exact value of *k_&mu_*). In other order of things, the interconnect resistance is related to temperature by [14]:

(7) $$R(T)=R_{0}[1+\alpha_{R}(T-T_{0})]$$

where *R*_0_ is the resistance at room temperature, and *α_r_* is the temperature coefficient—e.g., 0.004308 and 0.00401 for Al and Cu respectively. Interconnect resistance increases linearly with increasing temperature and so does the propagation delay as yielded from Equation (1).

Taking the preceding equations into account, a reasonable approximation for the delay of the proposed sensor, *t_tot_*, as a function of the temperature, *T*, is the following:

(8) $$t_{\text{tot}}(T)\approx t_{\text{tot}0}[1+k_{1}(T-T_{0})]$$

where *k*_1_ is a process-dependent parameter and *t_tot_*_0_ is the total delay at room temperature. Experimental results will show the validity of this approximation.

All of the above models are based upon previous work presented and validated in the scientific literature, which, albeit can display certain inaccuracies, offer a good understanding on how delay chains will behave under temperature changes.

## 4. Sensor Characterization

The proposed sensor has been characterized in two different contexts: Under constant temperature, to validate the delay equations and structure, illustrated by the measurement of within-die variations; and under varying temperature, to obtain the characteristics of the sensor employed as a temperature sensor.

### 4.1. Constant-Temperature Characterization

With the first experiment we want to examine the linearity of Equation (8) for a constant temperature, analyzing the stability of *k*_1_. Given that this value is a process-dependent parameter, this delay measurement can be used to sense process variations, both die-to-die and within-die.

Two Digilent Basys 2 prototyping boards, mounting a Spartan-3E XC3S100E, were identically characterized, implementing two identical sensors on each of them (one on each of the edges of the die). The sizes of the counter and comparator were fixed, and different lengths of the sensing delay chain were used. Each stage in the delay chain is constructed utilizing both LUT and latch pairs available in a Spartan 3E slice. Table 2 shows implementation results for one of the sensors in terms of size and actual delay, and compares it with the topology proposed by [6] for temperature monitoring. As seen in the table, the proposed topology can obtain similar delays to other sensors, using fewer combinational resources and, significantly, without making use of any clock tree, a potentially scarce resource in programmable devices.

**Figure 4.**
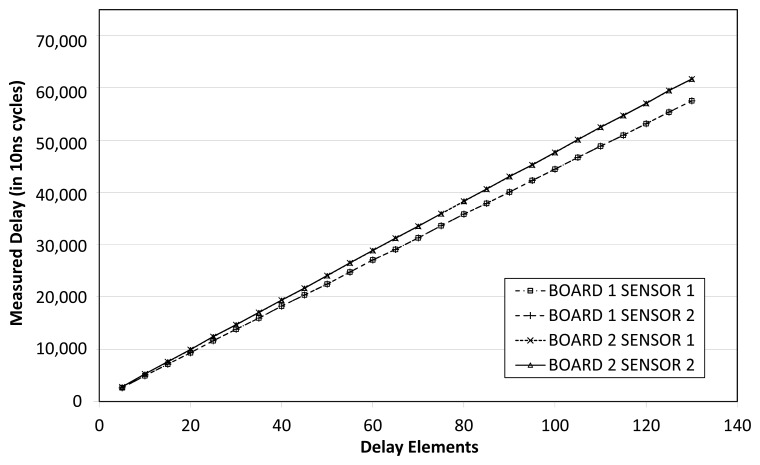
Constant-temperature sensor characterization.

In order to validate the linearity of Equation (8), the second experiment sets up sensors ranging from 5 to 130 delay stages, sampled with a 100 MHz time-to-digital counter on each board. In order to measure actual combinational delay of the sensor, the average of 200 measurements was taken for each sensor size and on each board. Figure 4 shows the actual delay of each sensor size, detailed for each board and for each embedded sensor. The linear regression curve is also included in the figure. At each point, the sensor incurs some error, *i.e.*, there are some discrepancies between the actual measured value and the ideal value given by the linear regression.

It can be seen that sensors on the same board offer an almost identical response, thus, it can be inferred that spatially-correlated intra-die process variations have little impact on these boards or that they are compensated somehow. Furthermore, the plot shows that board 1 is 6% faster than board 2.

**Figure 5.**
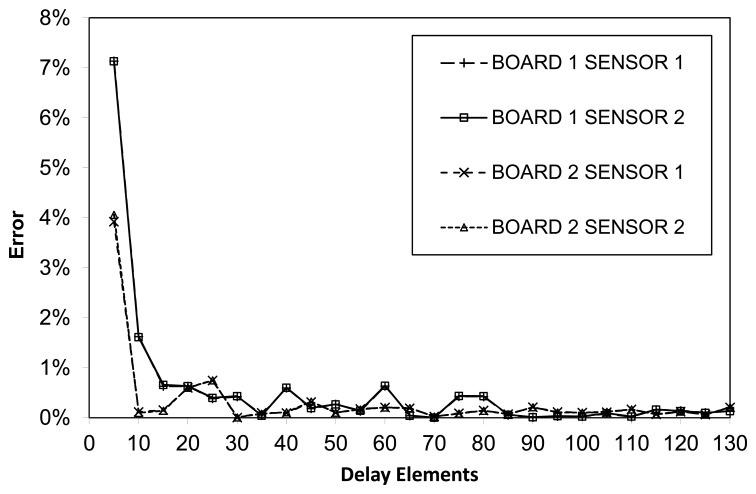
Constant-temperature measurement errors.

Figure 5 shows the relative error—the ratio between the error and the ideal measurement—for each point in the curve. Shorter combinational lengths incur a higher relative error. More precisely, no matter the sensor size, the error displays a constant deviation, implying a higher measurement error for small-sized sensors. This can be explained by taking into account the slight and constant delays introduced in the sensor by its internal control logic, which have a greater impact in smaller sensors. Consequently, there is a simple tradeoff between accuracy and sensor area so designers should insert sensors of at least a minimum number of delay stages. A closer observation of the graph shows the error coming down quickly from around 7.5% for 5 delay stages to 1.7% for 10 stages, and to 0.5% for 15 stages. At the same time there is little interest in increasing the size of the sensor, as the relative error will not improve past the 0.20% point (in our case for 40 delay stages and above). Hence for this particular device, designers should generate a total delay of 15 to 20 times that of a full slice (two pairs of LUT+latch) to obtain the best possible accuracy at the minimum cost in terms of area.

**Figure 6.**
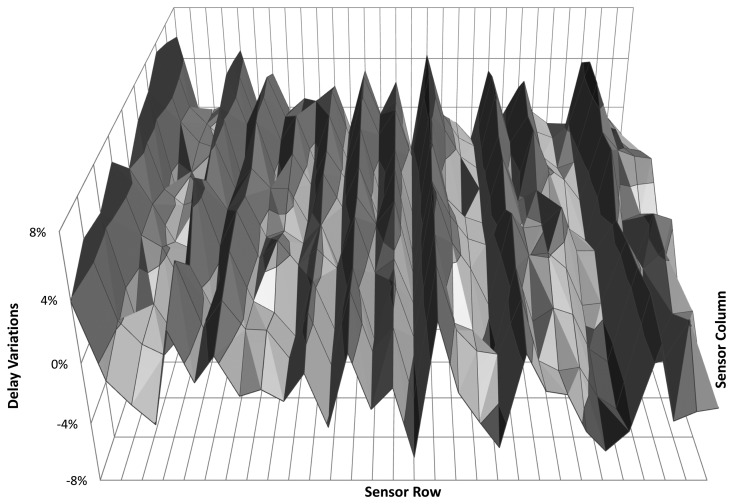
Virtex-5 process variability map. Delay variation as a function of the location in the Field Programmable Gate Arrays (FPGA) die.

As final experiment at constant temperature, the variability map of a larger FPGA has been extracted to illustrate the use of the proposed sensor with a different device and to measure within-die variations. The device under test was a 65 nm Virtex-5 LX50T from Xilinx, on a Digilent Genesys board. A full characterization of this device requires a dense mesh of sensors with a small granularity (short delay chains). The sensors are connected by means of the light-weight monitoring network proposed in [3], which employs a single on-chip time-to-digital converter to simultaneously perform all digitizations. Figure 6 shows the results for the whole FPGA measured from an array of 30 × 10 sensors, each built from 16 delay elements. The plot displays the variation around the average of all measured points. This experiment proves that the self-timed sensor accomplishes the task of process calibration in a very time-effective way. First, the clock deployment phase is skipped with the corresponding resource overhead reduction; second, it is very easy to adjust the trade-off between the delay chain length—sensor size—and the number of monitors which translates into sensor accuracy and spatial resolution, respectively; third, this process can be automated with little effort [15].

### 4.2. Temperature Variations

The second set of experiments has been carried out under varying temperature conditions to validate the use of the self-timed structure as a temperature sensor. A network of four temperature monitors has been implemented in a Spartan-3E XC3S100E mounted over a Digilent Basys 2 prototyping board. The sensors in the network have respectively 20, 40, 60 and 80 delay stages, to analyze the behavior of the sensor with different chain lengths.

**Figure 7.**
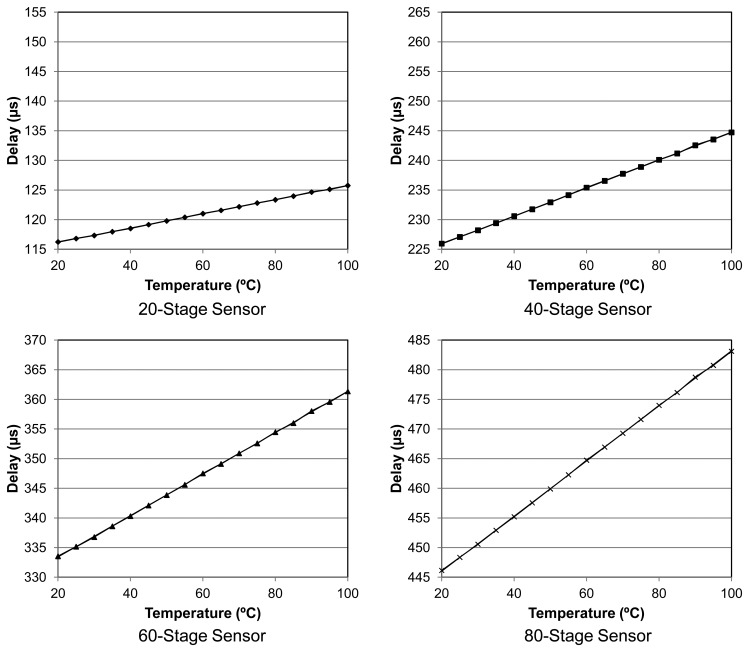
Delay *vs.* temperature transfer function for 20, 40, 60 and 80 stage sensors.

A stepper oven was used to cover the temperature range from 20 to 100 °C controlling the temperature with an external sensor of ± 0.1 °C accuracy. One hundred measurements per sensor were taken every 5 °C. Figure 7 displays the measurements for all four sensors, along with their respective linear regressions performed doing a two-point calibration. A good linear behavior in the transfer function is observed for the whole set of sensors. As expected, the slope is proportional to the number of delay elements proving the validity of Equation (8). In contrast to previous work [6], also based on delay chains, our sensors present a slope dependence on the number of elements because a fixed external clock is not used.

**Figure 8.**
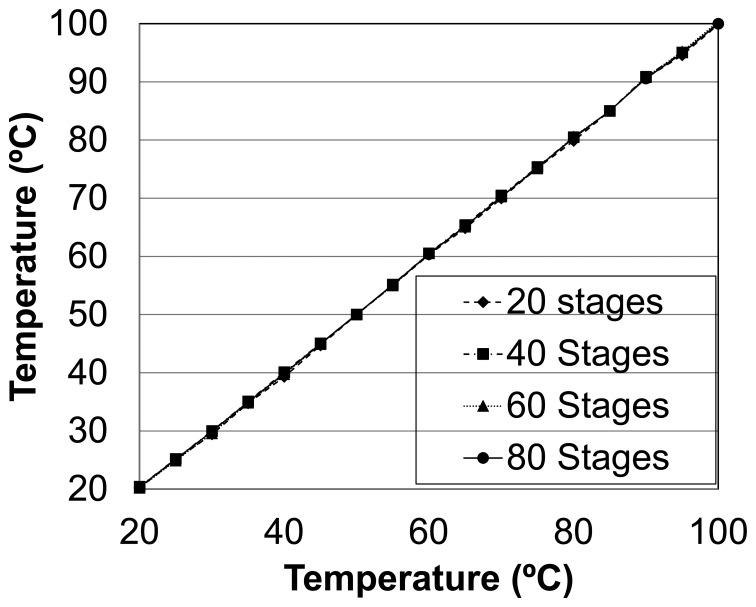
Temperature *vs.* temperature curve.

**Figure 9.**
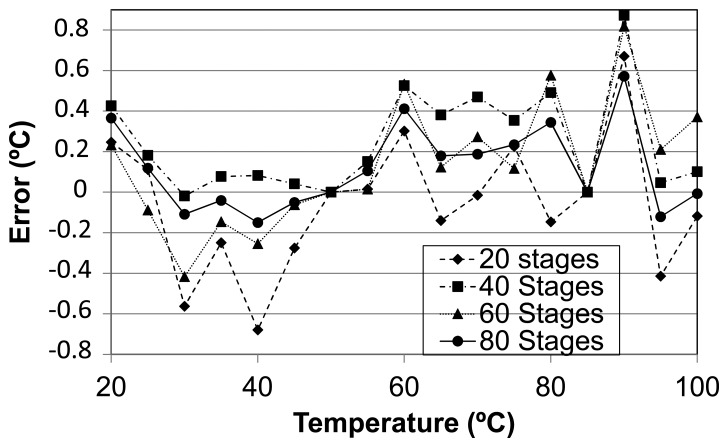
Temperature *vs.* temperature error.

The transfer function for the four sensors is very close to the ideal response (*i.e.*, the linear regression of two points) as shown in Figure 8, the error distribution for a two-point calibration is displayed in Figure 9. Each error is calculated as the difference between the measured value and the ideal one provided by the two-point linear regression. More precisely, Table 3 displays the maximum errors of different sized sensors. As can be seen, error figures for the same measurement are comparable across all sensors regardless of their size. The implication of this is that smaller sensors are preferable as long as the associated time-to-digital converter is sufficiently fast, the thermal sensitivity is constant for all sensor sizes, while the quantization error is reduced as the sensor size increases. Table 3 also compares the proposed sensor with different delay chain lengths to [6]. As shown for a bigger temperature interval, employing linear regression–instead of second order curvature—the proposed sensor improves the accuracy even for short delay chains.

## 5. Conclusions

This paper has introduced a new delay sensor for FPGAs. The sensor employs time amplification to generate a pulse whose width is dependent on the combinational delay of a chain of logic elements. It employs a feedback loop in asynchronous logic to build a structure that operates without the need of any external clock. This is translated into the simplification of the routing process and the avoidance of having to work with multiple clock signals. Employing the network structure presented in [3], several sensors can share a single time-to-digital converter, saving area and power overheads.

The sensor has been characterized both at constant and varying temperature, proving its capability to measure process and temperature fluctuations. Analytical equations for the sensor have been formulated and verified by the experimental results. As a temperature sensor, it has been measured to have an error of ± 0.67 °C, over the range of 20–100 °C, employing 20 logic elements with a 2-point calibration.

## Figures and Tables

**Table 1.** Clock generator truth table, *f*(*x*).

**Condition**	**Start**	**Done (sensor output)**	**Delay chain output**	**Clock generator output**
C1	0	0	0	0
C2	0	1	0	1
C3	0	0	1	0
C4	0	1	1	0
C5	1	0	0	1
C6	1	1	0	0
C7	1	0	1	0
C8	1	1	1	0

**Table 2.** Room-temperature implementation results.

**Parameter**	**Chen *et al.*** [6]	**Proposed**
External Clocks	1	0
Delay Elements (LUT + LATCH pairs)	75	80
Loop Count	4096	1024
Actual Delay (ns)	240,000	180.000
Total Area (LUT+D-FF Pairs)	140	110

**Table 3.** Varying temperature characterization results and comparison

**Sensor**	**Error (°C)**	**Range (°C)**	**Logic Elements**	**Calibration**
Chen *et al.* [6]	−1.5–0.8	0–75	75 (Altera ACEX 1K)	2-point, 2nd order curve
Proposed	− 0.67–0.67	20–100	20 (Xilinx Spartan-3E XC3S100E)	2-point, linear
Proposed	−0.02–0.87	20–100	40 (Xilinx Spartan-3E XC3S100E)	2-point, linear
Proposed	−0.41–0.81	20–100	60 (Xilinx Spartan-3E XC3S100E)	2-point, linear
Proposed	−0.12–0.57	20–100	80 (Xilinx Spartan-3E XC3S100E)	2-point, linear

## References

[1] Ghosh S., Roy K. (2010). Parameter variation tolerance and error resiliency: New design paradigm for the nanoscale era. Proc. IEEE.

[2] Zick K.M., Hayes J.P. (2012). Low-cost sensing with ring oscillator arrays for healthier reconfigurable systems. ACM Trans. Reconfigurable Technol. Syst..

[3] Ituero P., Lopez-Vallejo M., Marcos M., Osuna C. (2012). Light-weight on-chip monitoring network for dynamic adaptation and calibration. Sens. J. IEEE.

[4] Long J., Memik S.O., Memik G., Mukherjee R. (2008). Thermal monitoring mechanisms for chip multiprocessors. ACM Trans. Archit. Code Optimi..

[5] Kumar A. (2010). CAD Techniques for Robust FPGA Design Under Variability. Ph.D Thesis.

[6] Chen P., Shie M.C., Zheng Z.Y., Zheng Z.F., Chu C.Y. (2007). A fully digital time-domain smart temperature sensor realized with 140 FPGA logic elements. Circuits Syst. I Regul. Pap. IEEE Trans..

[7] Zick K.M., Hayes J.P. (2010). On-Line Sensing for Healthier FPGA Systems.

[8] Franco J., Boemo E., Castillo E., Parrilla L. Ring Oscillators as Thermal Sensors in FPGAs: Experiments in Low Voltage.

[9] Hung E., Wilton S., Yu H., Chau T., Leong P.H.W. A Detailed Delay Path Model for FPGAs.

[10] Rabaey J.M., Chandrakasan A., Nikolic B. (2003). Digital Integrated Circuits.

[11] Sedcole P., Cheung P.Y.K. Within-Die Delay Variability in 90nm FPGAs and Beyond.

[12] Masuda H., Ohkawa S., Kurokawa A., Aoki M. Challenge: Variability Characterization and Modeling for 65- to 90-nm Processes.

[13] Friedberg P., Cao Y., Cain J., Wang R., Rabaey J., Spanos C. Modeling Within-Die Spatial Correlation Effects for Process-Design Co-Optimization.

[14] Wolpert D., Ampadu P. (2012). Managing Temperature Effects in Nanoscale Adaptive Systems.

[15] Gómez-Osuna C., Sánchez M.A., Ituero P., López-Vallejo M. A Monitoring Infrastructure for FPGA Self-Awareness Dynamic Adaptation.

